# Therapeutic Evaluation of Intra-articular Platelet-Rich Plasma, Steroid, and Viscosupplement Injections for Sacroiliitis: A Retrospective Comparative Study

**DOI:** 10.7759/cureus.105097

**Published:** 2026-03-12

**Authors:** Pasala Jayavardhan Yadav, Gils Thampi, Nagakumar J S

**Affiliations:** 1 Orthopaedics, Sri Devaraj Urs Medical College (SDUMC), Kolar, IND

**Keywords:** corticosteroid injection, hyaluronic acid, oswestry disability index, platelet-rich plasma, sacroiliac joint, sacroiliitis, viscosupplementation, visual analog scale

## Abstract

Introduction

Sacroiliitis is a well-recognized cause of axial low back pain and significantly contributes to chronic cases seen in orthopedic and pain clinics. While intra-articular corticosteroid injections provide established short-term symptom relief, their long-term effectiveness remains variable. Platelet-rich plasma (PRP) has emerged as a biological alternative with regenerative potential, and viscosupplementation using hyaluronic acid offers viscoelastic and anti-inflammatory effects that may enhance joint function. This study aimed to compare the therapeutic efficacy of intra-articular corticosteroid, PRP, and viscosupplement injections in treating sacroiliitis.

Materials and methods

This retrospective comparative study included 60 adults with clinically and magnetic resonance imaging (MRI)-confirmed sacroiliitis who were treated between January 2022 and December 2024. Patients received one of the following treatments: fluoroscopy-guided intra-articular injection of 40 mg methylprednisolone acetate combined with lidocaine (steroid group; n=20), 5 mL of autologous PRP prepared using a standardized double-spin centrifugation protocol (PRP group; n=20), or intra-articular hyaluronic acid viscosupplementation (viscosupplement group; n=20). Pain and functional status were assessed using the Visual Analog Scale (VAS) and Oswestry Disability Index (ODI) at baseline, one week, one month, three months, and six months. Statistical analysis was conducted using repeated measures and one-way analysis of variance (ANOVA).

Results

Baseline VAS scores were comparable among the steroid (7.9±0.8), PRP (7.8±0.9), and viscosupplement (7.8±0.7) groups (p>0.05). The steroid group demonstrated greater early pain reduction at one week (4.6±0.9) compared to the PRP (6.2±0.8) and viscosupplement (5.8±0.9) groups. At six months, the PRP group showed the greatest improvement, with a mean VAS of 3.8±1.0, followed by the viscosupplement group (4.3±1.1), while the steroid group had higher scores (5.1±1.2) (p<0.05). Similarly, the mean ODI at six months was lowest in the PRP group (31.9±6.8%), followed by the viscosupplement (35.6±7.2%) and steroid groups (40.3±7.5%) (p<0.05). All groups showed significant improvement from baseline (p<0.001). No major complications were observed.

Conclusion

Intra-articular injections of corticosteroids, PRP, and viscosupplements effectively reduce pain and disability in sacroiliitis. Corticosteroids provide rapid early symptom relief, while PRP demonstrates superior mid-term improvement. Viscosupplementation offers sustained clinical benefits and represents a safe and effective therapeutic alternative.

## Introduction

Sacroiliitis, defined as inflammation of the sacroiliac joint, accounts for approximately 15-30% of chronic low back pain cases evaluated in specialty clinics [1,2]. The sacroiliac joint plays a critical biomechanical role in load transfer between the spine and lower extremities, and its dysfunction can significantly impair mobility and quality of life. Etiologies include inflammatory spondyloarthropathies, degenerative arthropathy, mechanical overload, trauma, and pregnancy-related pelvic instability [3]. Diagnosis relies on clinical provocative tests and magnetic resonance imaging (MRI) findings such as bone marrow edema, synovitis, and joint inflammation [4]. Image-guided intra-articular injections serve both diagnostic and therapeutic purposes and are commonly used in patients with persistent symptoms despite conservative management, including analgesics and physiotherapy [5]. Recent systematic reviews and meta-analyses have demonstrated that therapeutic sacroiliac joint injections can provide significant pain relief and functional improvement in appropriately selected patients [6].

Corticosteroids provide potent anti-inflammatory effects and have demonstrated effectiveness in reducing pain and improving function in sacroiliac joint disorders. However, their clinical benefits may diminish over time, and repeated use can be associated with local tissue damage and systemic complications [7]. Platelet-rich plasma (PRP) contains concentrated autologous growth factors that modulate inflammatory cascades, enhance tissue healing, and improve joint homeostasis [8]. Randomized and comparative studies on sacroiliac and lumbar facet joint pathologies have shown that corticosteroids offer faster early pain relief, whereas PRP provides more sustained clinical improvement at intermediate follow-up [9,10]. Similar sustained benefits of PRP have been reported in knee osteoarthritis, facet arthropathy, and other degenerative musculoskeletal conditions [11-14].

Viscosupplementation with hyaluronic acid represents an additional therapeutic approach that may enhance sacroiliac joint function. Hyaluronic acid is a key component of synovial fluid, contributing to joint lubrication, viscoelastic shock absorption, and the modulation of inflammatory mediators [15]. Intra-articular injections of hyaluronic acid have demonstrated clinical efficacy in osteoarthritis of synovial joints, including the knee and facet joints, by improving biomechanics and reducing pain [16]. Emerging evidence suggests that viscosupplementation may provide sustained symptom relief in sacroiliac joint dysfunction through mechanical stabilization and anti-inflammatory effects, with a favorable safety profile compared to repeated corticosteroid injections [17].

Despite growing interest in biologic and viscoelastic therapies, comparative clinical data evaluating corticosteroids, PRP, and viscosupplementation for sacroiliitis remain limited, particularly within the Indian population. Therefore, this study aimed to compare the clinical and functional outcomes of fluoroscopy-guided intra-articular injections of corticosteroids, PRP, and viscosupplements in patients with sacroiliitis. The assessment was conducted using the Visual Analog Scale (VAS) for pain and the Oswestry Disability Index (ODI) for functional evaluation over a six-month follow-up period.

## Materials and methods

This retrospective comparative study was conducted in the Department of Orthopaedics at R.L. Jalappa Hospital, Tamaka, Kolar, India, from January 2022 to December 2024, following institutional ethical clearance from the Central Ethics Committee of Sri Devaraj Urs Academy of Higher Education and Research (approval number: SDUAHER/R&D/CEC/SDUMC-PG/348/NF/-2025-26). Medical records of adult patients diagnosed with sacroiliitis who underwent fluoroscopy-guided sacroiliac joint injections during the study period were reviewed. Inclusion criteria comprised patients aged 18-65 years with clinically suspected sacroiliitis confirmed by MRI findings, such as bone marrow edema, synovitis, or erosive changes. Diagnosis was supported by localized sacroiliac tenderness and at least two positive provocative tests, including the FABER (Flexion, Abduction, and External Rotation) test, Gaenslen's test, or thigh thrust test [18,19]. All patients had failed conservative management for a minimum of six weeks, including analgesics, activity modification, and supervised physiotherapy. Patients with prior sacroiliac injections within six months, active infection, pregnancy, coagulopathy, systemic inflammatory flare requiring biologic therapy, or significant lumbar spine pathology necessitating surgical intervention were excluded.

As this was a retrospective study, treatment allocation was not randomized. The choice of injection therapy, that is, corticosteroid, PRP, or viscosupplement, was determined by the treating physician based on clinical judgment, patient preference, symptom severity, and the availability of biologic therapies at the time of treatment.

A total of 60 eligible patients were identified and divided into three equal groups (n=20 each) based on the type of injection received. Group A (steroid group) received an intra-articular injection of 40 mg methylprednisolone acetate combined with 2 mL of 2% lidocaine. Group B (PRP group) received 5 mL of autologous PRP prepared using a standardized double-spin centrifugation protocol (first spin at 1500 rpm for 10 minutes, followed by a second spin at 3500 rpm for 10 minutes) to obtain a concentrated platelet fraction. Although platelet concentration in the PRP preparation was not routinely quantified during the study period, the standardized centrifugation protocol was uniformly applied to all patients to obtain a platelet-enriched fraction. Group C (viscosupplement group) received intra-articular viscosupplementation with 2 mL of a hyaluronic acid preparation (20 mg), a viscoelastic agent designed to improve joint lubrication and modulate inflammation within synovial joints [20,21]. The viscosupplement used was a commercially available hyaluronic acid preparation routinely employed at our institution; however, specific molecular weight characteristics were not consistently recorded in the retrospective data. All procedures were performed by senior orthopedic consultants with more than five years of experience in spine and image-guided interventional procedures to ensure procedural consistency and minimize operator-dependent variability.

All injections were performed under strict aseptic conditions in the operating room using fluoroscopic guidance. Patients were positioned prone on a radiolucent operating table, with a pillow placed under the abdomen to reduce lumbar lordosis and facilitate optimal access to the sacroiliac joint. After sterile skin preparation with povidone-iodine solution and appropriate draping, local anesthetic infiltration was administered at the needle entry site. Using a posterior oblique approach under fluoroscopic visualization, a spinal needle was carefully advanced toward the inferior synovial portion of the sacroiliac joint. Correct intra-articular placement was confirmed by injecting a small amount of radiographic contrast, demonstrating appropriate intra-articular spread before administering the designated injectate. Careful attention was paid to avoid vascular or neural injury during needle advancement.

Following the procedure, patients were observed for a short period to monitor for immediate complications and were discharged on the same day with instructions to avoid strenuous physical activity for 24-48 hours. Patients were advised to gradually resume routine daily activities thereafter. No structured rehabilitation protocol or additional intra-articular treatments were administered during the follow-up period. Pain intensity was assessed using the VAS, a validated 0-10 numeric pain rating scale [22]. Functional disability was evaluated using the ODI Version 2.1a, a widely used and validated questionnaire for assessing low back pain [23]. Clinical assessments were recorded at baseline (pre-injection) and at one week, one month, three months, and six months post-injection. Because this study was based on a retrospective chart review, blinding of outcome assessors was not applicable. VAS and ODI scores were obtained from routine clinical follow-up records documented during patient visits.

The sample size was determined by the number of patients who met the eligibility criteria during the study period. Since this was a retrospective observational study, a formal prospective sample size calculation was not performed.

Statistical analysis

Data were entered into Microsoft Excel (Microsoft Corporation, Redmond, Washington, United States) and analyzed using IBM SPSS Statistics for Windows, Version 25.0 (IBM Corp., Armonk, New York, United States). Continuous variables are presented as mean±standard deviation (SD). Repeated measures analysis of variance (ANOVA) was used to evaluate intra-group changes over time, while one-way ANOVA was employed to compare continuous variables among the three treatment groups at each follow-up interval. Categorical variables were analyzed using the chi-squared test. A p-value of less than 0.05 was considered statistically significant.

## Results

All three groups were comparable at baseline. The mean age was 42.8±9.6 years in the steroid group, 41.2±10.3 years in the PRP group, and 43.1±8.9 years in the viscosupplement group (ANOVA; p=0.74). The male-to-female ratio was similar across groups (11:9 in the steroid group, 10:10 in the PRP group, and 12:8 in the viscosupplement group; chi-square; p=0.81). The mean duration of symptoms prior to intervention was 8.6±3.2 months, 8.9±3.6 months, and 9.1±3.4 months, respectively (ANOVA; p=0.88). Unilateral sacroiliac involvement was present in 70%, 75%, and 70% of patients in the steroid, PRP, and viscosupplement groups, respectively (chi-square; p=0.93). No statistically significant baseline differences were observed, confirming appropriate comparability between groups prior to intervention (Table 1).

**Table 1:** Baseline demographic and clinical characteristics (N=60) Values are presented as mean±standard deviation or as the number of patients (percentage), as appropriate. PRP: platelet-rich plasma

Parameter	Steroid (n=20)	PRP (n=20)	Viscosupplement (n=20)	Test statistic	P-value
Age (years)	42.8±9.6	41.2±10.3	43.1±8.9	F=0.31	0.74
Male/female	11/9	10/10	12/8	χ²=0.42	0.81
Symptom duration (months)	8.6±3.2	8.9±3.6	9.1±3.4	F=0.13	0.88
Unilateral involvement	14 (70%)	15 (75%)	14 (70%)	χ²=0.14	0.93
Bilateral involvement	6 (30%)	5 (25%)	6 (30%)	χ²=0.14	0.93

All three groups demonstrated a significant reduction in VAS scores from baseline (p<0.001). The steroid group showed rapid early improvement, with VAS scores decreasing from 7.9±0.8 at baseline to 4.6±0.9 at one week and 4.2±1.0 at one month, but increasing to 5.1±1.2 at six months, indicating a partial loss of effect. The PRP group exhibited gradual and sustained improvement, with VAS scores decreasing from 7.8±0.9 at baseline to 3.8±1.0 at six months. The viscosupplementation group showed sustained intermediate improvement, with VAS scores decreasing from 7.8±0.7 to 4.3±1.1 at six months. Intergroup analysis revealed superior early relief with steroids at one week (p<0.001), whereas PRP demonstrated significantly better outcomes at six months (p=0.001), with viscosupplementation showing consistent intermediate benefit (Table 2).

**Table 2:** Mean VAS scores over time (N=60) Values are expressed as the mean±standard deviation. The VAS ranges from 0 (no pain) to 10 (maximum pain). Intergroup comparisons were conducted using one-way ANOVA. * indicates statistically significant difference (p<0.05). PRP: platelet-rich plasma; VAS: Visual Analog Scale; ANOVA: analysis of variance

Time	Steroid (n=20)	PRP (n=20)	Viscosupplement (n=20)	Test statistic	P-value
Baseline	7.9±0.8	7.8±0.9	7.8±0.7	F=0.02	0.91
1 week	4.6±0.9	6.2±0.8	5.8±0.9	F=18.6	<0.001*
1 month	4.2±1.0	5.6±0.9	5.0±0.9	F=9.4	0.003*
3 months	4.8±1.1	4.1±0.9	4.4±1.0	F=3.4	0.04*
6 months	5.1±1.2	3.8±1.0	4.3±1.1	F=8.7	0.001*

ODI scores improved significantly in all three groups from baseline (repeated measures ANOVA; p<0.001). In the steroid group, ODI decreased from 55.6±7.4% at baseline to 41.2±6.8% at one month, but slightly increased to 40.3±7.5% at six months, indicating a partial decline in functional improvement. The PRP group demonstrated progressive and sustained improvement, with ODI decreasing from 56.1±7.8% at baseline to 31.9±6.8% at six months, representing the greatest functional recovery. The viscosupplement group also showed sustained improvement, with ODI decreasing from 55.8±7.2% at baseline to 35.6±7.2% at six months. Intergroup comparison using one-way ANOVA showed statistically significant differences at three months (p=0.04) and six months (p=0.001), with PRP demonstrating superior functional outcomes and viscosupplementation showing intermediate but sustained benefits (Table 3).

**Table 3:** Mean ODI scores over time (N=60) Values are expressed as mean±standard deviation. The ODI is reported as a percentage disability (0-100%), with lower scores indicating better functional outcomes. Intergroup comparisons were performed using one-way ANOVA. * indicates statistically significant difference (p<0.05). PRP: platelet-rich plasma; ODI: Oswestry Disability Index; ANOVA: analysis of variance

Time point	Steroid (n=20)	PRP (n=20)	Viscosupplement (n=20)	Test statistic (F)	P-value
Baseline	55.6±7.4	56.1±7.8	55.8±7.2	0.03	0.96
1 month	41.2±6.8	45.1±7.0	43.2±7.1	2.30	0.12
3 months	38.5±7.1	34.6±6.5	36.8±6.9	3.60	0.04*
6 months	40.3±7.5	31.9±6.8	35.6±7.2	9.80	0.001*

All three interventions were well-tolerated. Injection-site soreness occurred in two patients (10%) in the steroid group and in one patient each (5%) in the PRP and viscosupplement groups, with no statistically significant difference between groups (p=0.67). No infections, allergic reactions, or serious complications were observed (Table 4).

**Table 4:** Injection-related complications (N=60) Values are expressed as the number of patients (percentage). Categorical variables were compared using the chi-squared test. PRP: platelet-rich plasma; NA: not applicable due to the absence of events

Complication	Steroid (n=20)	PRP (n=20)	Viscosupplement (n=20)	Test statistic (χ²)	P-value
Injection-site soreness	2 (10%)	1 (5%)	1 (5%)	0.79	0.67
Infection	0	0	0	NA	NA
Allergic reaction	0	0	0	NA	NA

## Discussion

The present study demonstrated that intra-articular injections of corticosteroids, PRP, and viscosupplements all resulted in significant improvements in pain and functional outcomes in patients with sacroiliitis. The therapeutic role of image-guided sacroiliac joint injections has been supported by earlier systematic reviews demonstrating significant pain relief in selected patients with sacroiliac joint dysfunction [24]. Corticosteroids provided rapid early pain relief, consistent with their well-established anti-inflammatory mechanism of action [7]. However, a partial decline in clinical benefit was observed over time, aligning with findings from previous sacroiliac and lumbar injection studies [9]. This reduction in sustained efficacy likely reflects the temporary suppression of inflammatory mediators without addressing the underlying degenerative or biomechanical pathology. Furthermore, repeated corticosteroid exposure has been associated with potential chondrotoxic effects and soft tissue degeneration, which may limit long-term therapeutic use [7].

PRP demonstrated gradual but sustained improvement in both pain and functional scores, with superior outcomes at mid-term follow-up compared to corticosteroids. These findings are consistent with randomized trials involving the sacroiliac joint and lumbar facet joints, which reported greater durability of PRP compared to steroid injections [10,13]. Similar long-term benefits of PRP have been observed in knee osteoarthritis and other degenerative musculoskeletal conditions [11,12]. The delayed onset of symptom relief observed in the PRP group aligns with its biological mechanism, which involves growth factor-mediated modulation of inflammation, stimulation of tissue repair, and improvement of the joint microenvironment [8,17]. PRP has been shown to influence cytokine balance, promote angiogenesis, and enhance extracellular matrix remodeling, all of which contribute to sustained clinical benefits.

The viscosupplementation group demonstrated moderate but sustained improvements in both VAS and ODI scores. Hyaluronic acid plays a crucial role in maintaining synovial fluid viscosity, joint lubrication, and mechanical shock absorption while also exerting anti-inflammatory and chondroprotective effects [15]. Viscosupplementation has proven effective in alleviating pain and improving function in synovial joints such as the knee and facet joints, particularly in degenerative conditions [16,20]. The sustained improvement observed in this study may be attributed to enhanced joint biomechanics and a reduction in mechanical irritation within the sacroiliac joint. Compared to corticosteroids, viscosupplementation offers the advantage of avoiding potential steroid-related tissue damage while still providing meaningful functional improvement.

Comparatively, corticosteroids provided the most rapid early symptom relief, whereas PRP demonstrated the greatest sustained improvement at six months. Viscosupplementation showed consistent intermediate benefits, suggesting its potential role as an alternative therapeutic option, particularly for patients with degenerative sacroiliac pathology or those for whom repeated steroid exposure is undesirable. These findings support the expanding role of biologic and viscoelastic therapies in managing sacroiliac joint pain and align with emerging literature demonstrating the improved durability of regenerative and viscoelastic treatments compared to conventional corticosteroid therapy [12,21].

From a clinical perspective, corticosteroid injections remain appropriate when immediate symptom control is required. However, PRP may be preferable for patients seeking longer-lasting improvement and regenerative benefits, while viscosupplementation offers a safe and effective intermediate option with sustained functional gains. The significant improvements in both VAS and ODI scores across all three groups in this study support the role of image-guided intra-articular interventions in the comprehensive management of sacroiliitis.

Limitations

This study has several limitations. First, its retrospective design and single-center setting may limit the generalizability of the findings. Second, the sample size in each treatment group was modest, potentially reducing statistical power. Third, platelet concentration and the biological activity of PRP were not quantified, and individual variations in biological response may have influenced the outcomes. Fourth, imaging-based follow-up was not conducted to assess structural changes in the joint. Finally, follow-up was limited to six months, leaving longer-term outcomes unknown. Prospective, randomized, multicenter studies with larger sample sizes, standardized biologic preparation protocols, and extended follow-up periods are needed to better define the comparative effectiveness of corticosteroid, PRP, and viscosupplement injections in sacroiliitis.

## Conclusions

Intra-articular injections of corticosteroids, PRP, and viscosupplements significantly improved pain and functional outcomes in patients with sacroiliitis. Corticosteroids provided faster early pain relief, while PRP demonstrated the greatest sustained improvement at six months. Viscosupplementation showed consistent intermediate benefits with meaningful functional recovery. These findings support the use of image-guided biologic and viscoelastic therapies as safe and effective options for the individualized management of sacroiliac joint pain.

## References

[1] Cohen SP (2005). Sacroiliac joint pain: a comprehensive review of anatomy, diagnosis, and treatment. Anesth Analg.

[2] Szadek KM, van der Wurff P, van Tulder MW, Zuurmond WW, Perez RS (2009). Diagnostic validity of criteria for sacroiliac joint pain: a systematic review. J Pain.

[3] Slipman CW, Jackson HB, Lipetz JS, Chan KT, Lenrow D, Vresilovic EJ (2000). Sacroiliac joint pain referral zones. Arch Phys Med Rehabil.

[4] Maksymowych WP, Lambert RG, Østergaard M (2019). MRI lesions in the sacroiliac joints of patients with spondyloarthritis: an update of definitions and validation by the ASAS MRI working group. Ann Rheum Dis.

[5] Rupert MP, Lee M, Manchikanti L, Datta S, Cohen SP (2009). Evaluation of sacroiliac joint interventions: a systematic appraisal of the literature. Pain Physician.

[6] Janapala RN, Knezevic E, Knezevic NN (2023). Systematic review and meta-analysis of effectiveness of therapeutic sacroiliac joint injections. Pain Physician.

[7] Maugars Y, Mathis C, Berthelot JM, Charlier C, Prost A (1996). Assessment of the efficacy of sacroiliac corticosteroid injections in spondylarthropathies: a double-blind study. Br J Rheumatol.

[8] Alsousou J, Ali A, Willett K, Harrison P (2013). The role of platelet-rich plasma in tissue regeneration. Platelets.

[9] Kennedy DJ, Engel A, Kreiner DS, Nampiaparampil D, Duszynski B, MacVicar J (2015). Fluoroscopically guided diagnostic and therapeutic intra-articular sacroiliac joint injections: a systematic review. Pain Med.

[10] Chen AS, Solberg J, Smith C, Chi M, Lowder R, Christolias G, Singh JR (2022). Intra-articular platelet rich plasma vs corticosteroid injections for sacroiliac joint pain: a double-blinded, randomized clinical trial. Pain Med.

[11] Patel S, Dhillon MS, Aggarwal S, Marwaha N, Jain A (2013). Treatment with platelet-rich plasma is more effective than placebo for knee osteoarthritis: a prospective, double-blind, randomized trial. Am J Sports Med.

[12] Xiong Y, Gong C, Peng X (2023). Efficacy and safety of platelet-rich plasma injections for the treatment of osteoarthritis: a systematic review and meta-analysis of randomized controlled trials. Front Med (Lausanne).

[13] Baltzer AW, Enneper J, Baltzer LM, Godde G (2025). Platelet rich plasma for the therapy of the lumbar facet joint syndrome: a prospective study about CT-guided facet joint injections with PRP compared to local anesthetics. Orthop Rev (Pavia).

[14] Goodwin B, Averell N, Al-Shehab U, Ernazarov A, Price L, Choudhary A, Jermyn R (2023). Efficacy of platelet-rich plasma for sacroiliac joint dysfunction: a qualitative systematic review with pooled analysis. Regen Med.

[15] Altman RD, Manjoo A, Fierlinger A, Niazi F, Nicholls M (2015). The mechanism of action for hyaluronic acid treatment in the osteoarthritic knee: a systematic review. BMC Musculoskelet Disord.

[16] Henrotin Y, Raman R, Richette P (2015). Consensus statement on viscosupplementation with hyaluronic acid for the management of osteoarthritis. Semin Arthritis Rheum.

[17] Bowman S, Awad ME, Hamrick MW, Hunter M, Fulzele S (2018). Recent advances in hyaluronic acid based therapy for osteoarthritis. Clin Transl Med.

[18] Laslett M (2008). Evidence-based diagnosis and treatment of the painful sacroiliac joint. J Man Manip Ther.

[19] Dreyfuss P, Michaelsen M, Pauza K, McLarty J, Bogduk N (1996). The value of medical history and physical examination in diagnosing sacroiliac joint pain. Spine (Phila Pa 1976).

[20] Simopoulos TT, Manchikanti L, Gupta S (2015). Systematic review of the diagnostic accuracy and therapeutic effectiveness of sacroiliac joint interventions. Pain Physician.

[21] Singla V, Batra YK, Bharti N, Goni VG, Marwaha N (2017). Steroid vs. platelet-rich plasma in ultrasound-guided sacroiliac joint injection for chronic low back pain. Pain Pract.

[22] Hawker GA, Mian S, Kendzerska T, French M (2011). Measures of adult pain: Visual Analog Scale for Pain (VAS Pain), Numeric Rating Scale for Pain (NRS Pain), McGill Pain Questionnaire (MPQ), Short-Form McGill Pain Questionnaire (SF-MPQ), Chronic Pain Grade Scale (CPGS), Short Form-36 Bodily Pain Scale (SF-36 BPS), and Measure of Intermittent and Constant Osteoarthritis Pain (ICOAP). Arthritis Care Res (Hoboken).

[23] Fairbank JC, Pynsent PB (2000). The Oswestry Disability Index. Spine (Phila Pa 1976).

[24] McKenzie-Brown AM, Shah RV, Sehgal N, Everett CR (2005). A systematic review of sacroiliac joint interventions. Pain Physician.

